# Coexistence of muscle atrophy and high subcutaneous adipose tissue radiodensity predicts poor prognosis in hepatocellular carcinoma

**DOI:** 10.3389/fnut.2023.1272728

**Published:** 2023-10-06

**Authors:** Masatsugu Ohara, Goki Suda, Risako Kohya, Takashi Sasaki, Tomoka Yoda, Sonoe Yoshida, Qingjie Fu, Zijian Yang, Shunichi Hosoda, Osamu Maehara, Shunsuke Ohnishi, Yoshimasa Tokuchi, Takashi Kitagataya, Naoki Kawagishi, Masato Nakai, Takuya Sho, Mitsuteru Natsuizaka, Koji Ogawa, Naoya Sakamoto

**Affiliations:** ^1^Department of Gastroenterology and Hepatology, Hokkaido University Graduate School of Medicine, Sapporo, Japan; ^2^Laboratory of Molecular and Cellular Medicine, Faculty of Pharmaceutical Sciences, Hokkaido University, Sapporo, Japan

**Keywords:** low muscle mass, psoas muscles, skeletal muscle, liver disease, subcutaneous adipose tissue, muscle atrophy

## Abstract

**Introduction:**

We aimed to assess the prognostic implications of muscle atrophy and high subcutaneous adipose tissue (SAT) radiodensity in patients with hepatocellular carcinoma (HCC).

**Methods:**

In this retrospective study, muscle atrophy was assessed using the psoas muscle index (PMI) obtained from computed tomography. SAT radiodensity was evaluated based on radiodensity measurements. Survival and multivariate analyses were performed to identify factors associated with prognosis. The impact of muscle atrophy and high SAT radiodensity on prognosis was determined through survival analysis.

**Results:**

A total of 201 patients (median age: 71 years; 76.6% male) with HCC were included. Liver cirrhosis was observed in 72.6% of patients, and the predominant Child–Pugh grade was A (77.1%). A total of 33.3% of patients exhibited muscle atrophy based on PMI values, whereas 12.9% had high SAT radiodensity. Kaplan–Meier survival analysis demonstrated that patients with muscle atrophy had significantly poorer prognosis than those without muscle atrophy. Patients with high SAT radiodensity had a significantly worse prognosis than those without it. Muscle atrophy, high SAT radiodensity, the Barcelona Clinic Liver Cancer class B, C, or D, and Child–Pugh score ≥ 6 were significantly associated with overall survival. Further classification of patients into four groups based on the presence or absence of muscle atrophy and high SAT radiodensity revealed that patients with both muscle atrophy and high SAT radiodensity had the poorest prognosis.

**Conclusion:**

Muscle atrophy and high SAT radiodensity are significantly associated with poor prognosis in patients with HCC. Identifying this high-risk subgroup may facilitate the implementation of targeted interventions, including nutritional therapy and exercise, to potentially improve clinical outcomes.

## 1. Introduction

Hepatocellular carcinoma (HCC) is the fourth leading cause of cancer-related deaths, and its incidence has been increasing globally (1). Multiple prognostic factors are critical in the prognosis of HCC. These include the tumor status, as per the Barcelona Clinic Liver Cancer (BCLC) classification, liver function assessment according to the Child–Pugh score and albumin-bilirubin grade, and the patient’s general health status evaluated by the Eastern Cooperative Oncology Group performance status (2). While it is challenging to objectively assess a patient’s performance status, body composition, particularly muscle mass, offers an objective estimate of a patient’s physical, nutritional, and metabolic condition (3). Chronic liver diseases, including HCC, are known to cause secondary sarcopenia (4, 5); in addition, loss of muscle mass in patients with HCC is associated with a poorer prognosis and increased rates of recurrence (6–8). Moreover, in patients with chronic liver diseases, particularly those with liver cirrhosis (LC), the reported prevalence of sarcopenia is notably high and the loss of skeletal muscle mass progresses rapidly (7, 9–11). Therefore, it is crucial to assess muscle mass in patients with HCC to predict prognosis.

Recent studies suggest that aspects of body composition besides muscle mass also correlate with mortality in patients with decompensated cirrhosis and HCC (12). Factors such as subcutaneous adipose tissue (SAT) radiodensity (13, 14), intramuscular fat deposition, and visceral adiposity (15) have been reported as prognostic factors in patients with HCC. In particular, SAT radiodensity can be objectively measured by CT in Hounsfield units (HU), and average SAT radiodensity has been introduced as an indirect surrogate marker of adipose tissue quality (16). Consequently, the evaluation of body composition using CT, such as determining SAT radiodensity or detecting loss of skeletal muscle mass, is increasingly used as a non-invasive clinical tool with prognostic value. Importantly, early and appropriate identification of these abnormalities could enable interventions designed to rectify body composition abnormalities, potentially improving clinical outcomes (3). While a comprehensive analysis using SAT radiodensity and loss of skeletal muscle mass might predict prognosis in patients with HCC with greater accuracy, this approach is not yet fully clarified. In the present study, we aimed to evaluate the impact of muscle atrophy and SAT radiodensity on the prognosis of patients with HCC.

## 2. Materials and methods

In this retrospective study, we screened HCC patients who underwent proper CT imaging for evaluation of skeletal muscle mass and SAT radiodensity at Hokkaido University Hospital between July 2015 and May 2021. Patients were included if they were diagnosed with HCC according to Guidance by the American Association for the Study of Liver Diseases (17), had adequate clinical information and CT imaging for evaluation of skeletal muscle mass and SAT radiodensity, and were followed up for more than 3 months. We excluded patients with HCC who did not have adequate clinical and CT imaging information or were observed for a period of <3 months.

We collected the following clinical data: sex, age, etiology of chronic liver diseases, body mass index, blood test results, Child–Pugh class, TNM stage, BCLC class, presence of LC, and prognosis. In this study, LC was diagnosed based on liver biopsy, Fibroscan^®^ (Echosens, Paris, France) data, and/or radiologic findings, such as CT or magnetic resonance imaging, and laboratory data, as described in previous studies (18, 19). We investigated the factors associated with prognosis in patients with HCC, including skeletal muscle mass and SAT radiodensity by multivariate analysis. Subsequently, we analyzed the impact of skeletal muscle mass and SAT radiodensity on prognosis of these patients.

The study protocol conformed to the ethical guidelines of the Declaration of Helsinki and was approved by the Ethics Committee of Hokkaido University Hospital. All patients provided written informed consent to participate. This study was registered at the University Hospital Medical Information Network Clinical Trials Registry as UMIN 000030755.

### 2.1. Skeletal muscle mass calculation by computed tomography imaging

In the present study, to assess muscle atrophy, skeletal muscle mass was evaluated using the psoas muscle index (PMI). PMI was calculated from CT imaging as follows: the sum of the L3 level cross-sectional area of the right and left psoas muscle mass was identified by manual tracing and divided by height squared (cm^2^/m^2^) (18). Muscle atrophy was also defined according to our previous study as follows: PMI values <3.74 cm^2^/m^2^ for males, and < 2.29 cm^2^/m^2^ for females (20).

### 2.2. SAT radiodensity calculation by CT imaging

SAT radiodensity was measured using abdominal plain CT scans taken at the third lumbar vertebra (L3) levels. CT values were measured for regions of interests of four circles on subcutaneous fat away from major vessels. The mean values of these four regions of interests were used as the regions of interest of SAT radiodensity (21). High SAT radiodensity was also defined according to a previous study as follows: High SAT radiodensity > −74 HU for males and > −83 HU for females (14).

### 2.3. Endpoint

The primary endpoint of this study was to identify the factors associated with overall survival. Overall survival was defined as the duration from the time of inclusion until death. Patients who were still alive or transferred other hospitals were censored on the date of the last follow-up registered in the medical record.

### 2.4. Statistical analysis

Continuous variables were analyzed using the Mann–Whitney *U* test. Categorical variables were analyzed using the Fisher’s exact test.

Survival curves were constructed using the Kaplan–Meier method and compared using the log-rank test. Univariate Cox regression analysis was conducted for clinical factors and laboratory data; laboratory data that were included in the calculation of the Child–Pugh score and those that violated the proportional hazards assumption were excluded. Multivariate Cox regression analysis was conducted for factors showing significance (defined at *p* < 0.1) in the univariate analysis (22, 23). All *p*-values were two-tailed, and the level of significance was set at *p* < 0.05. All statistical data were generated using Prism 7.03 (GraphPad Software, Inc., La Jolla, CA) and EZR (Saitama Medical Center, Jichi Medical University, Saitama, Japan).

## 3. Results

### 3.1. Patient characteristics

**Figure 1:**
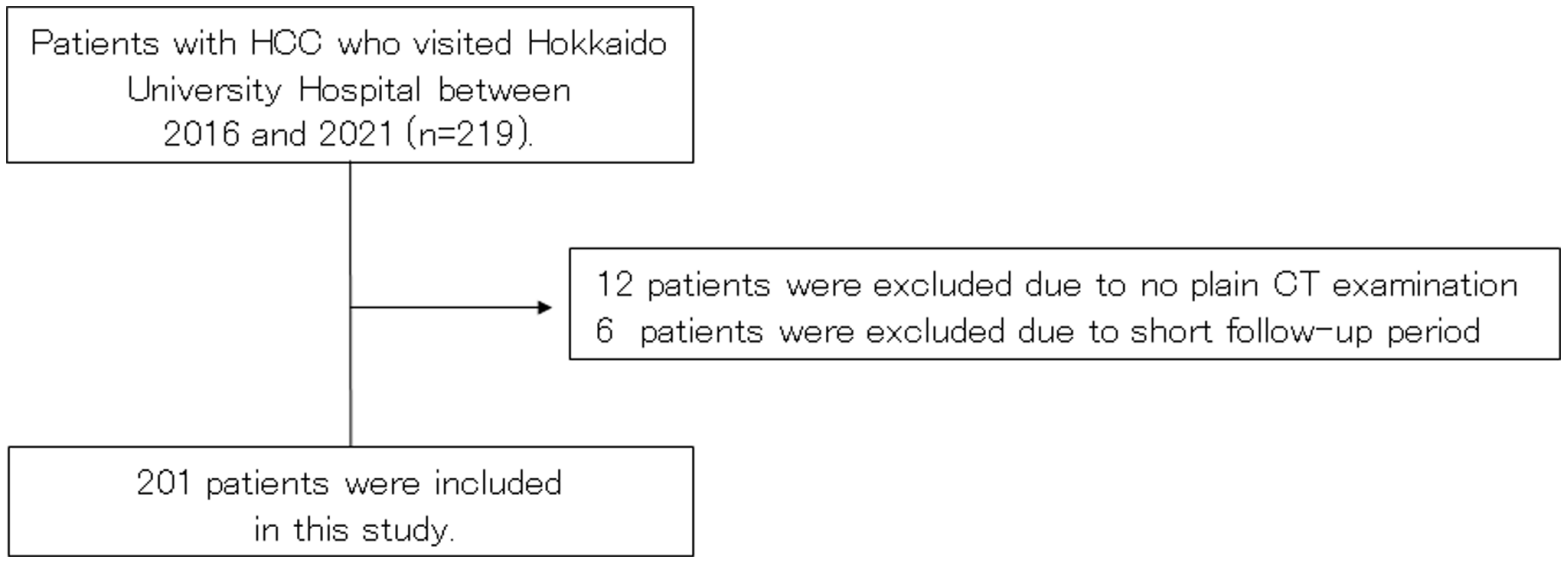
Patient flow chart. CT, computed tomography; HCC, hepatocellular carcinoma.

Patients with HCC who visited Hokkaido University Hospital between July 2015 and May 2021 were screened for inclusion. Ultimately, 201 patients met the inclusion criteria and were included in this study (Figure 1).

The baseline characteristics of included patients with HCC are shown in Table 1. The median age of patients was 71 years (range: 20–90 years), and 76.6% of them were males. A total of 72.6% had LC. A total of 77.1% had Child–Pugh A. A total of 31.3 and 30.3% had BCLC stage 0 and A. Based on the PMI values, 33.3% patients had muscle atrophy. The definition of high SAT radiodensity in previous reports was adopted (SAT > −74 HU for males and > −83 HU for females) (14), and a total of 12.9% patients had high SAT radiodensity.

**Table 1:** Baseline clinical and biochemical characteristics.

Variables	Overall (*n* = 201)
Age, years	71 (20–90)
Sex, male / female	154 (76.6%) / 47 (23.4%)
Etiology, HBV / HCV / NBNC	59 (29.4%) / 51 (25.4%) / 91 (45.2%)
Liver cirrhosis, + / −	146 (72.6%) / 55 (27.4%)
Stage, I / II / III / IV	67 (33.3%) / 59 (29.4%) / 45 (22.3%) / 30 (15.0%)
BCLC stage, 0 / A / B / C / D	63 (31.3%) / 61 (30.4%) / 43 (21.4%) / 28 (13.9%) / 6 (3.0%)
Child–Pugh grade, A / B / C	155 (77.1%) / 42 (20.9%) / 4 (2.0%)
Child–Pugh score	7 (5–11)
Follow-up period, years	2.84 (0.25–7.01)
PMI, cm^2^/m^2^	M: 4.15 (0.77–8.65)
	F: 2.81 (0.71–6.62)
Muscle atrophy, + / −	67 (33.3%) / 134 (66.7%)
SAT radiodensity, HU	M: −98.75 (−109.5 – −64.75)
	F: −96.62 (−114.75 – −58.75)
High SAT, + / −	26 (12.9%) / 175 (87.1%)
Body mass index, kg/m^2^	24.4 (14.9–36.8)
Ascites, + / −	40 (19.9%) / 161 (80.1%)
Platelet counts, x10^4^ /mm^3^	13.6 (3.00–42.4)
Prothrombin time, %	87.2 (19.5–125.4)
Serum albumin, g/dL	3.90 (2.3–4.90)
Aspartate transaminase, IU/L	37 (10–332)
Alanine aminotransferase, IU/L	26 (6–109)
Creatinine, mg/dL	0.79 (0.38–7.43)
Alpha-fetoprotein, ng/mL	6.4 (1.3–413503.0)

Data are presented as number, percentages of patients or median (range) values.

BCLC, the Barcelona Clinic Liver Cancer; F, female; HBV, hepatitis B virus; HCV, hepatitis C virus; NBNC, non-HBV non-HCV; PMI, psoas muscle mass index; M, male; SAT, subcutaneous adipose tissue.

### 3.2. Comparison of prognosis between HCC patients with and without muscle atrophy

**Figure 2:**
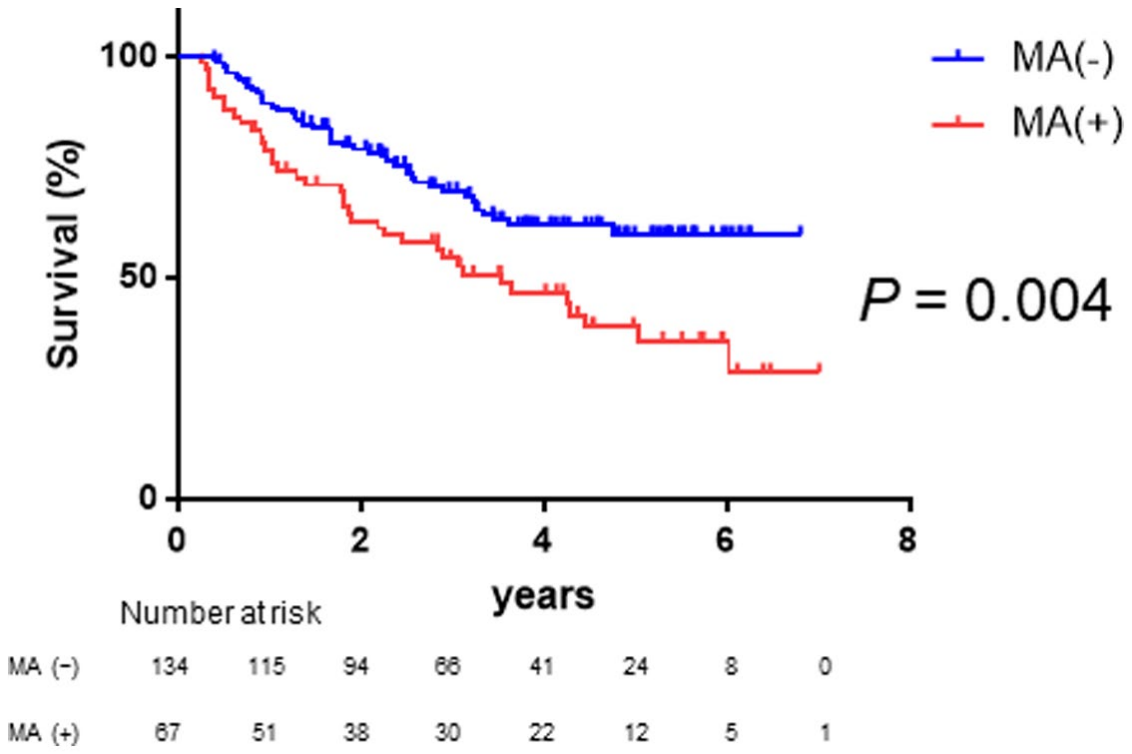
Survival rates in patients with and without muscle atrophy Kaplan–Meier estimates of overall survival stratified according to muscle atrophy in patients with HCC. The median overall survival is not reached in patients without muscle atrophy and 3.52 years in patients with muscle atrophy. Patients with muscle atrophy have a poorer prognosis than those without muscle atrophy (log-rank *p*-value = 0.004; crude Cox regression hazard ratio: 1.88, 95% confidence interval: 1.18–2.99). HCC, hepatocellular carcinoma.

As shown in Figure 2, Kaplan–Meier survival analysis revealed that patients with muscle atrophy had a significantly poorer prognosis than those without muscle atrophy (log-rank *p*-value = 0.004; crude Cox regression hazard ratio [HR]: 1.878, 95% confidence interval [CI]: 1.18–2.99). The comparison between characteristics of patient with or without muscle atrophy are shown in Table 2. Patients with muscle atrophy exhibited significantly higher rates of high SAT radiodensity and lower body mass index compared to those without muscle atrophy.

**Table 2:** Baseline clinical and biochemical characteristics of patients with or without muscle atrophy.

Variables	Without muscle atrophy (*N* = 134)	With muscle atrophy (*N* = 67)	*P*-values
Age, years	70 (20–88)	71 (44–90)	0.057
Sex, male / female	100 (74.6%) / 34 (25.4%)	54 (80.6%) / 13 (19.4%)	0.382
Etiology, HBV / HCV / NBNC	46 (34.3%) / 29 (21.7%) / 59 (44.0%)	13 (19.4%) / 22 (32.8%) / 32 (47.8%)	0.055
Liver cirrhosis, + / −	94 (70.1%) / 40 (29.9%)	52 (77.6%) / 15 (22.4%)	0.315
Stage, I / II / III / IV	49 (36.6%) / 37 (27.6%) / 27 (20.1%) / 21 (15.7%)	18 (26.9%) / 22 (32.8%) / 18 (26.9%) / 9 (13.4%)	0.559
BCLC stage, 0 / A / B / C / D	48 (35.8%) / 36 (26.9%) / 29 (21.7%) / 18 (13.4%) / 3 (2.2%)	15 (22.3%) / 25 (37.4%) / 14 (20.9%) / 10 (14.9%) / 3 (4.5%)	0.258
Child–Pugh grade, A / B / C	108 (80.6%) / 23 (17.2%) / 3 (2.2%)	47 (70.1%) / 19 (28.4%) / 1 (1.5%)	0.162
Child–Pugh score	5 (5–11)	6 (5–11)	0.051
Follow-up period, years	2.92 (0.39–6.81)	2.84 (0.25–7.01)	0.265
PMI, cm^2^/m^2^	M: 4.70 (3.75–8.65)	M: 2.94 (0.77–3.73)	<0.001
	F: 3.44 (2.30–6.62)	F: 0.71 (1.90–2.15)	<0.001
SAT radiodensity, HU	M: −100.25 (−114.75 – −62.0)	M: −93.25 (−110.75 – −58.75)	0.004
	F: −98.88 (−109.5 – −66.75)	F: −97.50 (−108.25 – −64.75)	0.803
High SAT, + / −	10 (7.5%) / 124 (92.5%)	16 (23.9%) / 51 (76.1%)	0.002
Body mass index, kg/m^2^	25.2 (16.3–36.8)	22.5 (14.9–36.1)	<0.001
Ascites, + / −	23 (17.2%) / 111 (82.8%)	17 (25.4%) / 50 (74.6%)	0.191
Platelet counts, x10^4^ /mm^3^	13.9 (4.4–40.0)	13.0 (3.0–42.4)	0.390
Prothrombin time, %	89.6 (19.5–123.30)	84.5 (19.6–125.4)	0.179
Serum albumin, g/dL	3.90 (2.3–4.90)	3.8 (2.3–4.8)	0.318
Aspartate transaminase, IU/L	34.5 (10–332)	38 (116–206)	0.259
Alanine aminotransferase, IU/L	26.5 (6–109)	25 (8–102)	0.860
Creatinine, mg/dL	0.79 (0.38–7.43)	0.80 (0.43–2.11)	0.863
Alpha-fetoprotein, ng/mL	5.9 (1.3–413503.0)	8.2 (1.5–316096.7)	0.176

Data are presented as number, percentages of patients or median (range) values.

BCLC, the Barcelona Clinic Liver Cancer; F, female; HBV, hepatitis B virus; HCV, hepatitis C virus; NBNC, non-HBV non-HCV; PMI, psoas muscle mass index; M, male; SAT, subcutaneous adipose tissue.

### 3.3. Comparison of prognosis in patients with HCC with and without high SAT radiodensity

**Figure 3:**
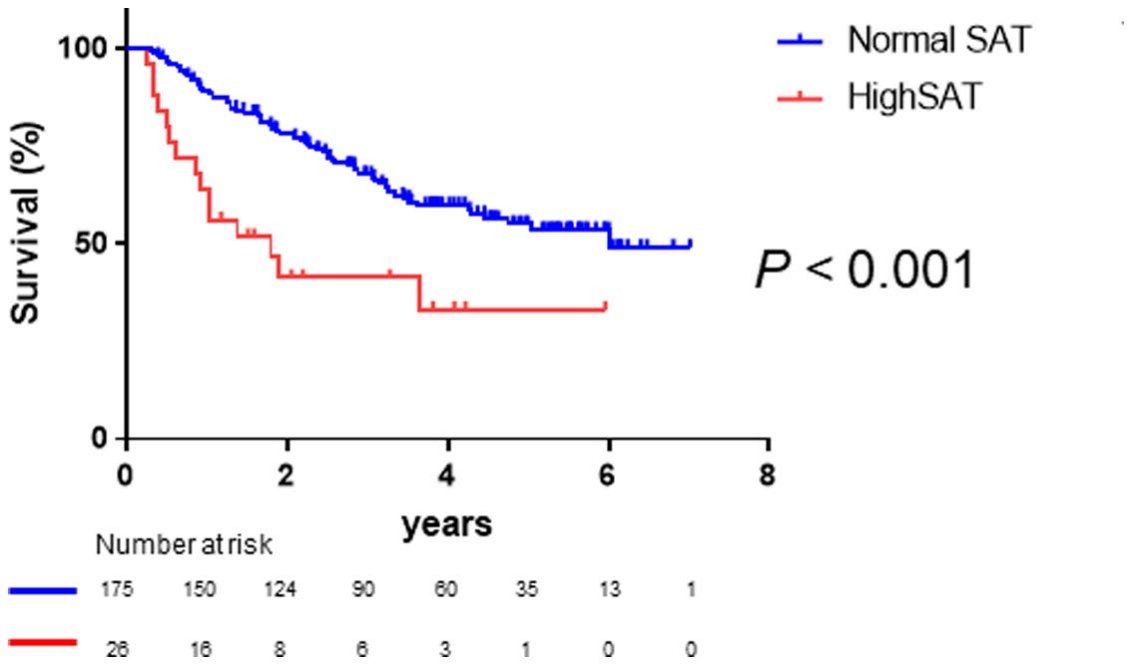
Survival rates in patients with normal and high subcutaneous adipose tissue (SAT) radiodensity Kaplan–Meier estimates of overall survival stratified according to SAT radiodensity in patients with HCC. The median overall survival is 6.02 years in patients with normal SAT radiodensity and 1.80 years in patients with high SAT radiodensity. Patients with high SAT radiodensity have a poorer prognosis than those with normal SAT radiodensity (log-rank *p*-value <0.001; crude Cox regression hazard ratio: 2.66, 95% confidence interval: 1.18–5.96). HCC, hepatocellular carcinoma; SAT, subcutaneous adipose tissue.

As shown in Figure 3, Kaplan–Meier survival analysis revealed that patients with high SAT radiodensity had a significantly poorer prognosis than those without it (log-rank *p*-value <0.001, crude Cox regression HR: 2.66, 95% CI: 1.18–5.96). Table 3 presents a comparison of characteristics between patients with and without high SAT radiodensity. Patients with high SAT radiodensity were significantly more likely to be male, have muscle atrophy and ascites, display lower body mass index, have reduced albumin levels, exhibit lower PMI in males, and have a worse Child–Pugh grade compared to those without it.

**Table 3:** Baseline clinical and biochemical characteristics of patients with normal SAT or high SAT.

Variables	Normal SAT (*N* = 175)	High SAT (*N* = 26)	*P*-values
Age, years	70 (20–90)	71 (41–88)	0.330
Sex, male / female	130 (74.3%) / 45 (25.7%)	24 (92.3%) / 2 (7.7%)	0.047
Etiology, HBV / HCV / NBNC	51 (29.1%) / 42 (24.0%) / 82 (46.9%)	8 (30.8%) / 9 (34.6%) / 9 (34.6%)	0.389
Liver cirrhosis, + / −	125 (71.4%) / 50 (28.6%)	21 (80.8%) / 5 (19.2%)	0.479
Stage, I / II / III / IV	60 (34.2%) / 51 (29.2%) / 38 (21.7%) / 26 (14.9%)	7 (26.9%) / 8 (30.8%) / 7 (26.9%) / 4 (15.4%)	0.946
BCLC stage, 0 / A / B / C / D	57 (32.6%) / 54 (30.8%) / 34 (19.4%) / 26 (14.9%) / 4 (2.3%)	6 (23.1%) / 7 (26.9%) / 9 (34.6%) / 2 (7.7%) / 2 (7.7%)	0.177
Child–Pugh grade, A / B / C	141 (80.6%) / 31 (17.7%) / 3 (1.7%)	14 (53.9%) / 11 (42.3%) / 1 (3.8%)	0.012
Child–Pugh score	5 (5–11)	6 (5–11)	0.002
Follow-up period, years	3.08 (0.31–7.01)	1.28 (0.25–5.96)	<0.001
Psoas muscle mass, cm^2^/m^2^	M: 4.26 (0.77–8.65)	M: 3.31 (1.28–7.59)	0.007
	F: 2.81 (0.71–6.62)	F: 3.48 (1.79–5.17)	0.874
Muscle atrophy, + / −	51 (29.1%) / 124 (70.9%)	16 (61.5%) / 10 (38.5%)	0.002
SAT radiodensity, HU	M: −100.25 (−114.75 – −83.00)	M: −73.38 (−82.25 – −58.75)	<0.001
	F: −99.0 (−109.50 – −80.5)	F: −65.75 (−66.75 – −64.75)	0.002
Body mass index, kg/m^2^	24.9 (14.9–36.8)	21.2 (15.1–36.0)	<0.001
Ascites, + / −	27 (15.4%) / 148 (84.6%)	13 (50.0%) / 13 (50.0%)	<0.001
Platelet counts, x10^4^ /mm^3^	13.6 (3.00–40.0)	13.95 (5.1–42.4)	0.631
Prothrombin time, %	87.9 (19.5–125.4)	84.0 (42.0–122.2)	0.206
Serum albumin, g/dL	3.90 (2.30–4.90)	3.50 (2.30–4.20)	<0.001
Aspartate transaminase, IU/L	37 (10–332)	39 (16–139)	0.427
Alanine aminotransferase, IU/L	26 (7–109)	24 (6–77)	0.770
Creatinine, mg/dL	0.79 (0.38–7.43)	0.82 (0.48–2.56)	0.291
Alpha-fetoprotein, ng/mL	6.4 (1.3–413503.0)	6.95 (1.5–316096.7)	0.631

Data are presented as number, percentages of patients or median (range) values.

BCLC, the Barcelona Clinic Liver Cancer; F, female; HBV, hepatitis B virus; HCV, hepatitis C virus; NBNC, non-HBV non-HCV; PMI, psoas muscle mass index; M, male; SAT, subcutaneous adipose tissue.

### 3.4. Univariate and multivariate Cox regression analysis regarding clinical factors associated with overall survival

Subsequently, we analyzed the clinical factors associated with overall survival in patients with HCC. Multivariate regression analysis was performed using variables of clinical factors with *p* < 0.1 in the univariate analyses, which were sex and age. Multivariate regression analysis revealed that muscle atrophy (HR, 1.69; 95% CI, 1.09–2.63; *p* = 0.019), high SAT radiodensity (HR, 2.30; 95% CI, 1.23–4.31; *p* = 0.01), and BCLC class B, C, or D (HR, 4.53; 95% CI, 2.76–7.43; *p* < 0.001), and Child–Pugh score ≥ 6 (HR, 1.71; 95% CI, 1.02–2.88; *p* = 0.043) were significantly associated with overall survival in patients with HCC (Table 4).

**Table 4:** Prognostic factors in patients with HCC.

Variables	Univariate analysis		Multivariate analysis	
	Hazard ratio	*P*-values	Hazard ratio	*P*-values
Age, >70 years old	1.03 (0.67–1.59)	0.884		
Sex, male	1.01 (0.62–1.66)	0.961		
Liver cirrhosis, +	1.58 (0.92–2.69)	0.095	1.25 (0.69–2.26)	0.460
Child–Pugh score, ≥6	2.84 (1.82–4.43)	<0.001	1.71 (1.02–2.88)	0.043
ALT, >26 IU/L	1.72 (1.11–2.66)	0.016	1.40 (0.88–2.22)	0.160
Creatinine, > 0.79 mg/dL	1.33 (0.87–2.05)	0.193		
Plt, < 13.6 × 10^4^ / mm^3^	1.14 (0.74–1.75)	0.556		
BCLC stage, B, C, and D	5.31 (3.37–8.37)	<0.001	4.53 (2.76–7.43)	<0.001
Muscle atrophy, +	1.88 (1.22–2.90)	0.004	1.69 (1.09–2.63)	0.019
High SAT, +	2.70 (1.53–4.75)	0.001	2.30 (1.23–4.31)	0.010
Ascites, +	2.23 (1.36–3.67)	0.002	1.03 (0.58–1.83)	0.930

ALT, alanine aminotransferase; BCLC, the Barcelona Clinic Liver Cancer; Plt, platelet counts SAT, subcutaneous adipose tissue.

### 3.5. Comparison of overall survival among the four classified groups based on muscle atrophy and high SAT radiodensity

**Figure 4:**
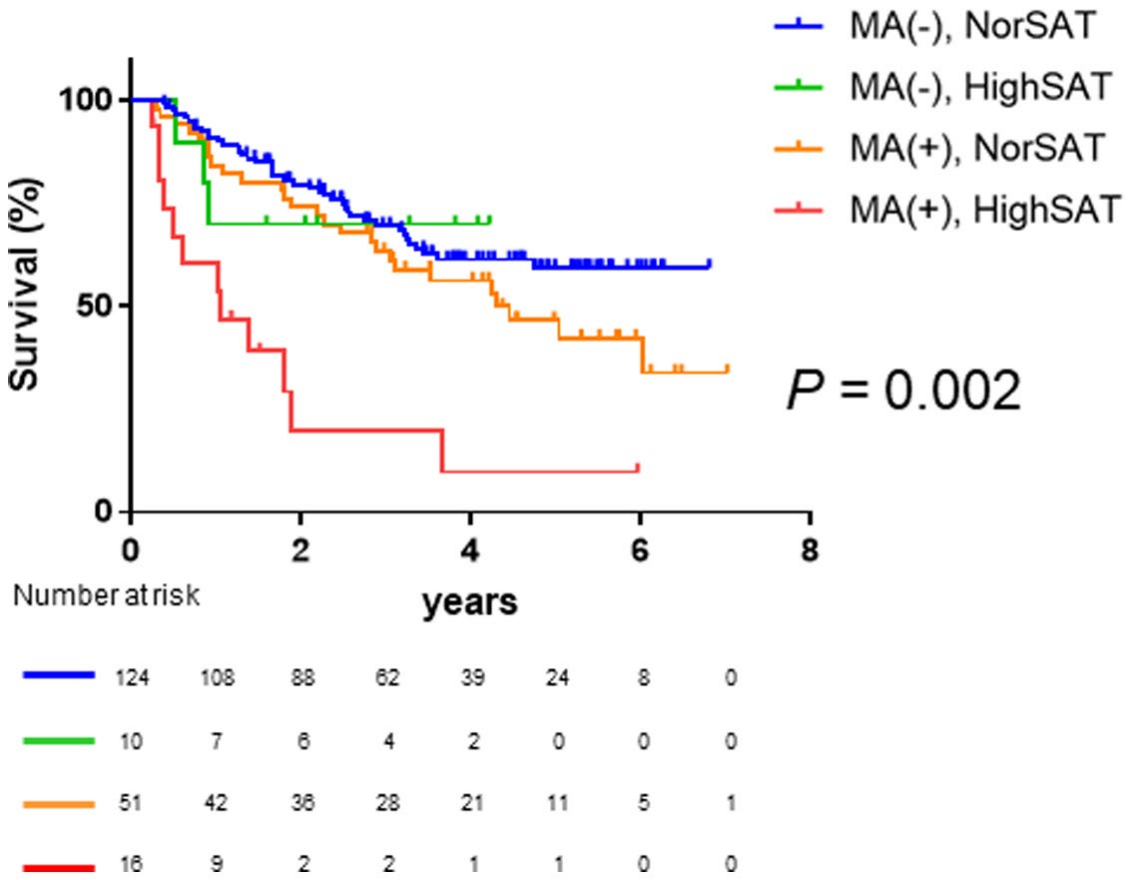
Comparison of overall survival among the four classified groups based on muscle atrophy and SAT radiodensity. The median overall survival is not reached in patients without muscle atrophy and normal SAT radiodensity and patients without muscle atrophy and high SAT radiodensity; it is 4.30 years in patients with muscle atrophy and normal SAT radiodensity and 1.04 years in patients with muscle atrophy and high SAT radiodensity. Overall survival is significantly different among the four classified groups based on muscle atrophy and SAT radiodensity (*p* = 0.002). HCC, hepatocellular carcinoma; SAT, subcutaneous adipose tissue.

Given that high SAT radiodensity and muscle atrophy were found to be significant and independent prognostic factors in patients with HCC, we further classified the patients into four groups based on the presence or absence of muscle atrophy and high SAT radiodensity. As shown in Figure 4, Kaplan–Meier survival analysis revealed that the overall survival among four groups was significantly different. In particular, the patients with muscle atrophy and high SAT radiodensity had a poor prognosis (median overall survival in the patients without muscle atrophy and with or without high SAT radiodensity was undefined, 4.29 years in the patients with muscle atrophy and without high SAT radiodensity, and 1.04 years in the patients with muscle atrophy and high SAT radiodensity; *p* = 0.002).

### 3.6. Stratified analysis

**Figure 5:**
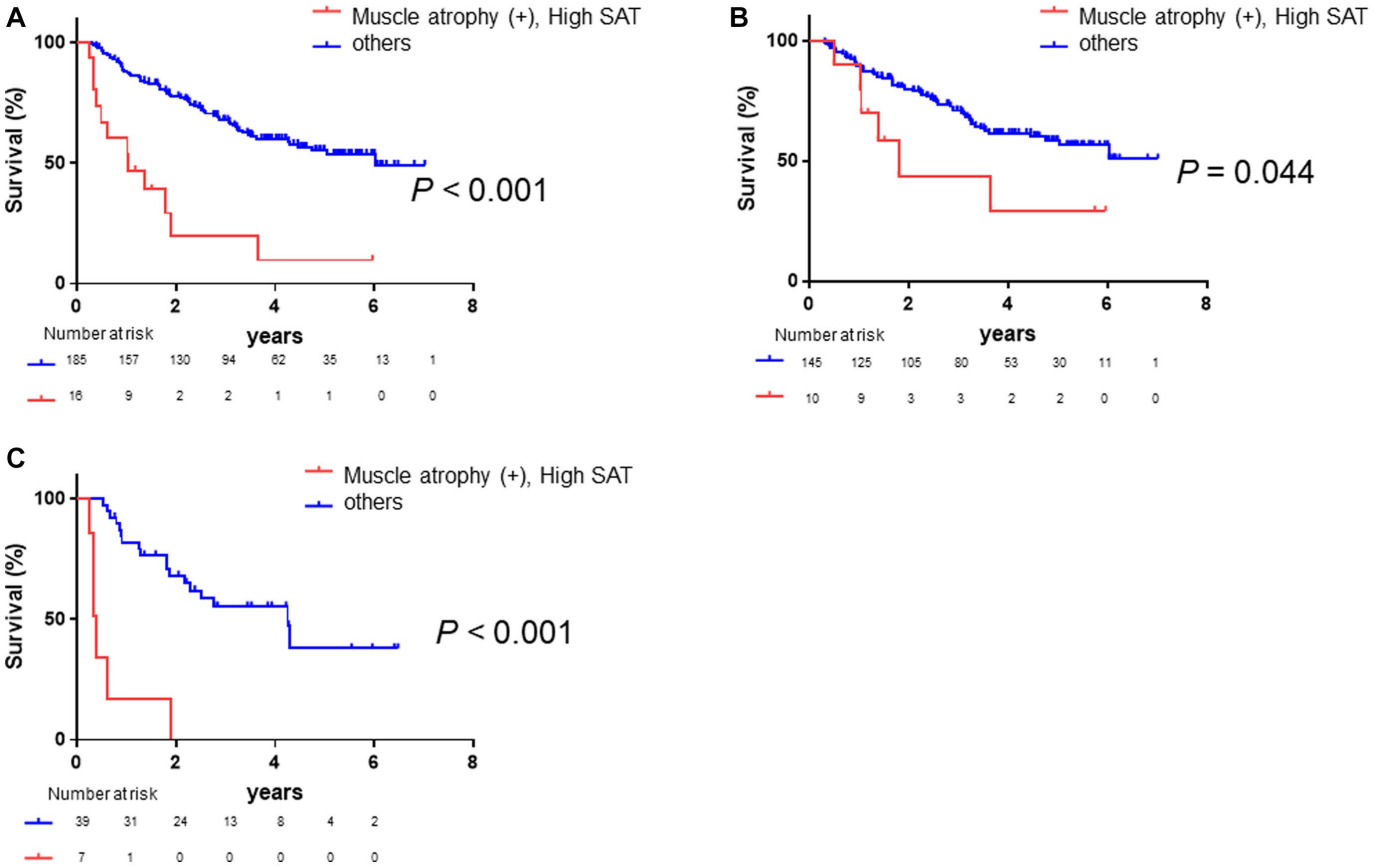
Survival rates in all patients and in those stratified by Child–Pugh grade. **(A)** Kaplan–Meier analysis is performed to stratify patients with and without muscle atrophy and high SAT radiodensity. The median overall survival is 1.04 years in patients with both muscle atrophy and high SAT radiodensity and 6.02 years in the other patients. Patients with muscle atrophy and high SAT radiodensity have a poorer prognosis than the other patients (log-rank *p* < 0.001; crude Cox regression HR: 4.53, 95% CI: 1.43–14.4). **(B)** Among patients with Child–Pugh grade A, Kaplan–Meier analysis is performed to stratify patients with and without muscle atrophy and high SAT radiodensity. The median overall survival is 1.79 years in patients with both muscle atrophy and high SAT radiodensity and not reached in the other patients. Patients with both muscle atrophy and high SAT radiodensity have a poorer prognosis than the other patients (log-rank *p*-value = 0.044; crude Cox regression HR: 3.48, 95% CI: 1.03–11.70). **(C)** Among the patients with Child–Pugh grade B or C, Kaplan–Meier analysis is performed to stratify patients with and without muscle atrophy and high SAT radiodensity. The median overall survival is 0.40 years in patients with both muscle atrophy and high SAT radiodensity and 4.25 years in the other patients. Patients with both muscle atrophy and high SAT radiodensity have a significantly poorer prognosis than the other patients (log-rank *p*-value <0.001; crude Cox regression HR: 10.43, 95% CI: 3.76–28.93). CI, confidence interval; HCC, hepatocellular carcinoma; HR, hazard ratio; SAT, subcutaneous adipose tissue.

Based on the results presented above (Figure 4), we assessed the prognosis in the entire population, distinguishing between patients with both muscle atrophy and high SAT radiodensity, and those without both before conducting stratified analysis. Patients with both muscle atrophy and high SAT radiodensity had a significantly poorer prognosis than the other patients (log-rank *p*-value <0.001, Figure 5A; crude Cox regression HR: 4.53, 95% CI: 1.43–14.4). Subsequently, we conducted subgroup analyses based on clinical factors contributing to overall survival of patients with HCC. First, we performed the subgroup analysis stratified with Child–Pugh grade A, B, and C. A total of 77.1% patients had Child–Pugh grade A, and 22.9% had Child–Pugh grade B or C. Among the patients with Child–Pugh grade A, the patients with muscle atrophy tended to have a poor prognosis (log-rank *p*-value = 0.054, Supplementary Figure 2A; crude Cox regression HR: 1.66, 95% CI: 0.95–2.90), and the patients with high SAT radiodensity had a similar tendency toward a poorer prognosis (log-rank *p*-value = 0.116, Supplementary Figure 2B; crude Cox regression HR: 1.86, 95% CI: 0.67–5.14). Patients with both muscle atrophy and high SAT radiodensity had a significantly poorer prognosis than the other patients (log-rank *p*-value = 0.044, Figure 5B; crude Cox regression, HR: 3.48 95% CI: 1.03–11.70). Among the patients with Child–Pugh grade B or C, the patients with muscle atrophy tended to have a poorer prognosis (log-rank *p*-value = 0.054, Supplementary Figure 2C; crude Cox regression HR: 2.17, 95% CI: 0.95–4.91), and the patients with high SAT radiodensity had a significantly poorer prognosis (log-rank *p*-value = 0.001, Supplementary Figure 2D; crude Cox regression HR: 3.61, 95% CI: 1.06–12.29). Similar to patients with Child–Pugh grade A, patients with both muscle atrophy and high SAT radiodensity had a significantly poorer prognosis than other patients with Child–Pugh grade B or C (log-rank *p*-value <0.001, Figure 5C; crude Cox regression, HR: 10.43, 95% CI: 3.76–28.93).

**Figure 6:**
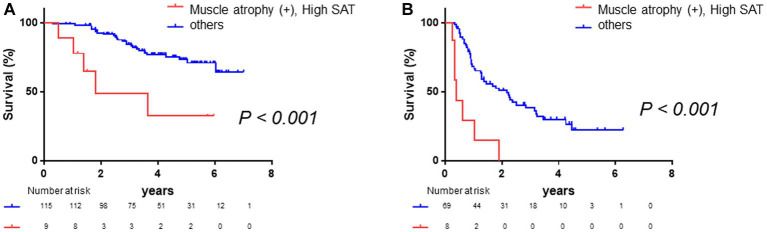
Survival rates stratified according to BCLC class. **(A)** Among patients with BCLC class 0 or A, Kaplan–Meier analysis is performed to stratify patients with and without muscle atrophy and high SAT radiodensity. The median overall survival is 1.80 years in patients with both muscle atrophy and high SAT radiodensity and not reached in the other patients. Patients with muscle atrophy and with high SAT radiodensity have a poorer prognosis than the other patients (log-rank *p*-value <0.001; crude Cox regression HR: 4.51, 95% CI: 1.72–11.85). **(B)** Among patients with BCLC class B, C, or D, Kaplan–Meier analysis was performed to stratify patients with and without muscle atrophy and high SAT radiodensity. The median overall survival is 0.40 years in patients with both muscle atrophy and high SAT radiodensity and 2.18 years in the other patients. Patients with both muscle atrophy and high SAT radiodensity have a poorer prognosis than the other patients (log-rank *p*-value <0.001; crude Cox regression HR: 5.36, 95% CI: 2.34–12.28). CI, confidence interval; HCC, hepatocellular carcinoma; HR, hazard ratio; SAT, subcutaneous adipose tissue.

Subsequently, we conducted a subgroup analysis based on BCLC class. A total of 61.7% of patients were BCLC class 0 or A. Among these patients, those with muscle atrophy had a significantly poorer prognosis (log-rank *p*-value = 0.005, Supplementary Figure 3A; crude Cox regression HR: 2.95, 95% CI: 1.38–6.30), and the patients with high SAT radiodensity had a poorer prognosis (log-rank *p*-value = 0.02, Supplementary Figure 3B; crude Cox regression HR: 2.98, 95% CI: 1.13–7.84). In addition, the patients with both muscle atrophy and high SAT radiodensity had a significantly poorer prognosis than the other patients (log-rank *p*-value <0.001, Figure 6A; crude Cox regression HR: 4.51, 95% CI: 1.72–11.85). Afterward, among the patients with BCLC class B, C, or D, the patients with muscle atrophy had a tendency toward a poor prognosis (log-rank *p*-value = 0.13, Supplementary Figure 3C; crude Cox regression HR: 1.53, 95% CI: 0.84–2.75), and the patients with high SAT radiodensity had a poorer prognosis (log-rank *p*-value = 0.006, Supplementary Figure 3D; crude Cox regression HR: 2.59, 95% CI: 1.28–5.21). Patients with both muscle atrophy and high SAT radiodensity had a significantly poorer prognosis than the other patients (log-rank *p*-value <0.001, Figure 6B; crude Cox regression HR: 5.36, 95% CI: 2.34–12.28).

**Figure 7:**
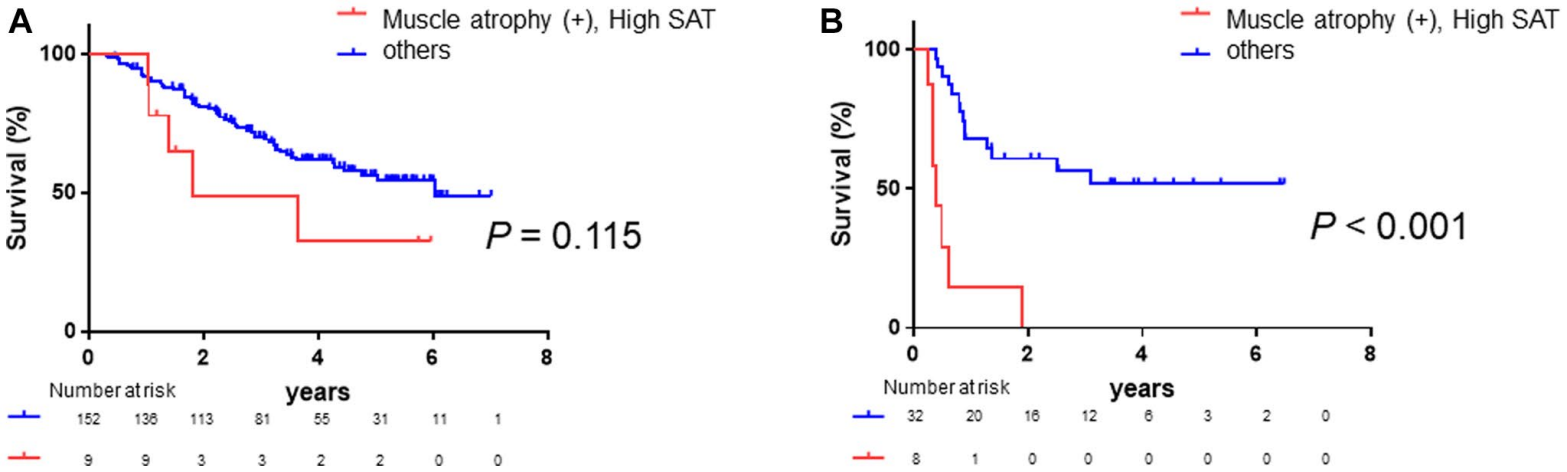
Survival rates stratified according to fluid retention **(A)** Among patients without ascites or pleural effusion, Kaplan–Meier analysis is performed to stratify patients with and without muscle atrophy and high SAT radiodensity. The median overall survival is 1.80 years in patients with both muscle atrophy and high SAT radiodensity and 6.03 years in the other patients. Patients with both muscle atrophy and with high SAT radiodensity tend to have a poorer prognosis than the other patients (log-rank *p*-value = 0.115; crude Cox regression HR: 2.05, 95% CI: 0.58–7.19). **(B)** Among patients with ascites or pleural effusion, Kaplan–Meier analysis is performed to stratify patients with and without muscle atrophy and high SAT radiodensity. The median overall survival is 0.40 years in patients with both muscle atrophy and high SAT radiodensity and not reached in the other patients. Patients with both muscle atrophy and with high SAT radiodensity have a poorer prognosis than the other patients (log-rank *p*-value <0.001; crude Cox regression HR: 6.55, 95% CI: 1.24–34.7). CI, confidence interval; HCC, hepatocellular carcinoma; HR, hazard ratio; SAT, subcutaneous adipose tissue.

Finally, we conducted a stratified subgroup analysis based on the presence of ascites or pleural effusion. A total of 19.9% of patients had ascites or pleural effusion. Among the 161 patients (80.1%) without ascites, patients with muscle atrophy had a poorer prognosis (log-rank *p*-value = 0.038, Supplementary Figure 4A; crude Cox regression HR: 1.70, 95% CI: 1.02–2.81), and the patients with high SAT radiodensity had a poorer prognosis (log-rank *p*-value = 0.049, Supplementary Figure 4B; crude Cox regression HR: 2.99, 95% CI: 1.01–8.88). Patients with both muscle atrophy and high SAT radiodensity had a tendency toward a poorer prognosis than other patients (log-rank *p*-value = 0.115, Figure 7A; crude Cox regression HR: 2.05, 95% CI: 0.58–7.19). Similarly, among patients with ascites or pleural effusion, patients with muscle atrophy had a poorer prognosis (log-rank *p*-value = 0.041, Supplementary Figure 4C; crude Cox regression HR: 2.40, 95% CI: 1.01–5.7), and patients with high SAT radiodensity had a tendency toward a poor prognosis (log-rank *p*-value = 0.091, Supplementary Figure 4D; crude Cox regression HR: 2.08 95% CI: 0.75–5.77). Patients with both muscle atrophy and high SAT radiodensity had a poorer prognosis than the others (log-rank *p*-value <0.001, Figure 7B; crude Cox regression HR: 6.55, 95% CI: 1.24–34.7).

## 4. Discussion

In this study, we demonstrated that muscle atrophy and high SAT radiodensity could significantly and independently predict prognosis in patients with HCC. Moreover, we highlighted the importance of evaluating both muscle atrophy and high SAT radiodensity as prognostic factors in patients with HCC because coexistence of muscle atrophy and high SAT radiodensity could predict quite poor prognosis in patients with HCC. Identifying this high-risk subgroup may facilitate the implementation of targeted interventions, including nutritional therapy and exercise, to potentially improve clinical outcomes.

Prognosis in patients with HCC is reported to be associated with functional liver reserve, TNM staging, BCLC class, and performance status. In addition to these factors, loss of skeletal muscle mass is reported to be an important factor associated with prognosis in patients with HCC (8, 24–26). Furthermore, the importance of assessing adipose tissue-related parameters such as intramuscular fat, visceral fat, and SAT radiodensity for predicting prognosis has been recently reported (12–15, 27–29). Among these studies, Ebadi et al. conducted a study on cirrhotic patients, half of which had HCC, and reported that SAT density could stratify prognosis. Moreover, Ebadi et al. suggested that morphological rearrangements of SAT, diagnosed by SAT density, might occur prior to the loss of SAT mass (14). In the present study, we found that HCC patients with high SAT radiodensity had significantly worse prognosis, which was consistent with the results of previous studies.

SAT plays a pivotal role in lipid storage and energy homeostasis and is now recognized for its multifaceted endocrine function, with adipokines playing a role in the regulation of diverse metabolic and inflammatory processes (30). High SAT radiodensity not only indicates a reduced SAT volume but also implies remodeling of adipose tissue, characterized by morphological changes such as atrophy, evidenced by smaller and shrunken adipocytes, expanded interstitial space, and infiltration of mononuclear cells. Therefore, it is suggested that high SAT radiodensity reflects the severe energy depletion associated with cirrhosis, leading to unfavorable clinical outcomes (14).

This study highlighted that multivariate analysis revealed both muscle atrophy and high SAT radiodensity as significant and independent poor prognostic factors. Furthermore, the coexistence of muscle atrophy and high SAT radiodensity was found to be a strong and significant predictor of poor prognosis in patients with HCC. To date, there have been limited studies performing simultaneous analysis of skeletal muscle mass and adipose tissue-related parameters as prognostic factors in HCC. Lim et al. reported that muscle depletion combined with visceral adiposity was a crucial prognostic factor for predicting survival in older patients with HCC who underwent transarterial chemoembolization (27). Additionally, Saeki et al. demonstrated that the absence of muscle depletion combined with a high visceral fat area could serve as a novel biomarker for patients with advanced HCC treated with sorafenib (28). Thus, combining assessment tools for muscle mass- and adipose tissue-related parameters may provide highly accurate stratification of HCC mortality risk. In patients with gastric cancer, subcutaneous but not visceral adipose tissue is a predictive marker of prognosis (31). This study is the first to demonstrate stratification of HCC prognosis based on a combination of muscle atrophy and SAT radiodensity. Furthermore, among Child–Pugh A patients, prognosis could not be stratified by the presence of muscle atrophy or high SAT radiodensity alone; however, the combination of muscle atrophy and SAT radiodensity proved effective in stratifying patients with poor prognosis. Therefore, simultaneous evaluation of muscle atrophy and high SAT radiodensity may hold clinical significance.

In patients with BCLC class B or more, muscle atrophy could not predict prognosis, whereas high SAT radiodensity could stratify prognosis. The reason why muscle atrophy could not predict the prognosis in those patients remains unclear; however, there is a hypothesis. In patients with BCLC class B or more, muscle mass had already decreased due to advanced HCC, and thus, prognosis could be stratified using only SAT radiodensity. In addition, the combination of muscle atrophy and SAT radiodensity more clearly stratified the prognosis. It has been reported that adipose tissue remodeling is observed at the early stages of cachexia (14). Thus, this study’s observation might reflect the presence of cancer-related cachexia, which results in poor prognosis.

Skeletal muscle mass is measured using bioelectrical impedance analysis (BIA), CT imaging with specialized software, CT imaging using a simple CT method and PMI calculation, and dual-energy X-ray absorptiometry. Among these, CT is commonly used for diagnostic purposes, recurrence screening, and evaluation of treatment response in patients with HCC (2, 17, 32). Our recent study highlighted that, for an accurate evaluation of relative changes in skeletal muscle mass in patients with chronic liver diseases, including HCC, CT imaging is a suitable method compared to BIA (33). In addition, CT imaging allows for the simultaneous evaluation of SAT radiodensity and muscle area. Therefore, CT imaging is a valuable tool not only for assessing HCC but also for evaluating the nutritional status of patients with HCC. Additionally, even when muscle mass was assessed using the simple method (*n* = 145) (34), which is a simpler CT-based method than PMI, patients with both muscle atrophy and high SAT had a poor prognosis (log-rank *p*-value <0.001, Supplementary Figure 1; crude Cox regression HR: 4.79, 95% CI: 1.48–15.5).

Our study had several limitations that need to be acknowledged. First, it was a retrospective study conducted at a single center. In addition, due to the retrospective nature of the study, certain clinical factors were either lacking or incomplete. Therefore, further validation of our results via a prospective multicenter study is required.

In conclusion, muscle atrophy and high SAT radiodensity are significantly associated with poor prognosis in patients with HCC. Our findings highlight the importance of evaluating muscle atrophy and high SAT radiodensity as prognostic factors in patients with HCC. This study is the first to report the significant negative impact of both muscle atrophy and high SAT radiodensity on survival in patients with HCC. In particular, for patients with muscle atrophy and high SAT radiodensity, interventions such as nutritional therapy and exercise may have the potential to improve their clinical outcomes.

## Data availability statement

The raw data supporting the conclusions of this article will be made available by the authors, without undue reservation.

## Ethics statement

The studies involving humans were approved by the Ethics Committee of Hokkaido University Hospital. The studies were conducted in accordance with the local legislation and institutional requirements. The participants provided their written informed consent to participate in this study.

## Author contributions

MO: Conceptualization, Data curation, Formal analysis, Investigation, Methodology, Project administration, Writing – original draft, Writing – review & editing. GS: Conceptualization, Funding acquisition, Software, Supervision, Visualization, Writing – review & editing. RK: Investigation, Writing – review & editing. TSa: Investigation, Writing – review & editing. TY: Investigation, Writing – review & editing. SY: Investigation, Writing – review & editing. QF: Investigation, Writing – review & editing. ZY: Investigation, Writing – review & editing. SH: Investigation, Writing – review & editing. OM: Investigation, Writing – review & editing. SO: Supervision, Writing – review & editing. YT: Investigation, Methodology, Writing – review & editing. TK: Investigation, Writing – review & editing. NK: Investigation, Writing – review & editing. MaN: Investigation, Methodology, Project administration, Writing – review & editing. TSh: Investigation, Methodology, Writing – review & editing. MiN: Supervision, Writing – review & editing. KO: Methodology, Supervision, Writing – review & editing. NS: Funding acquisition, Supervision, Writing – review & editing.
