# Supplementary material for: Coexistence of muscle atrophy and high subcutaneous adipose tissue radiodensity predicts poor prognosis in hepatocellular carcinoma
Source: Front Nutr. 2023 Oct 6;10:1272728. doi: 10.3389/fnut.2023.1272728 (PMC10587397; doi:10.3389/fnut.2023.1272728)
Supplement: Supplementary file 1 [file Data_Sheet_1.docx]

Supplementary Material

# Supplementary Figures

## Supplementary Figure 1
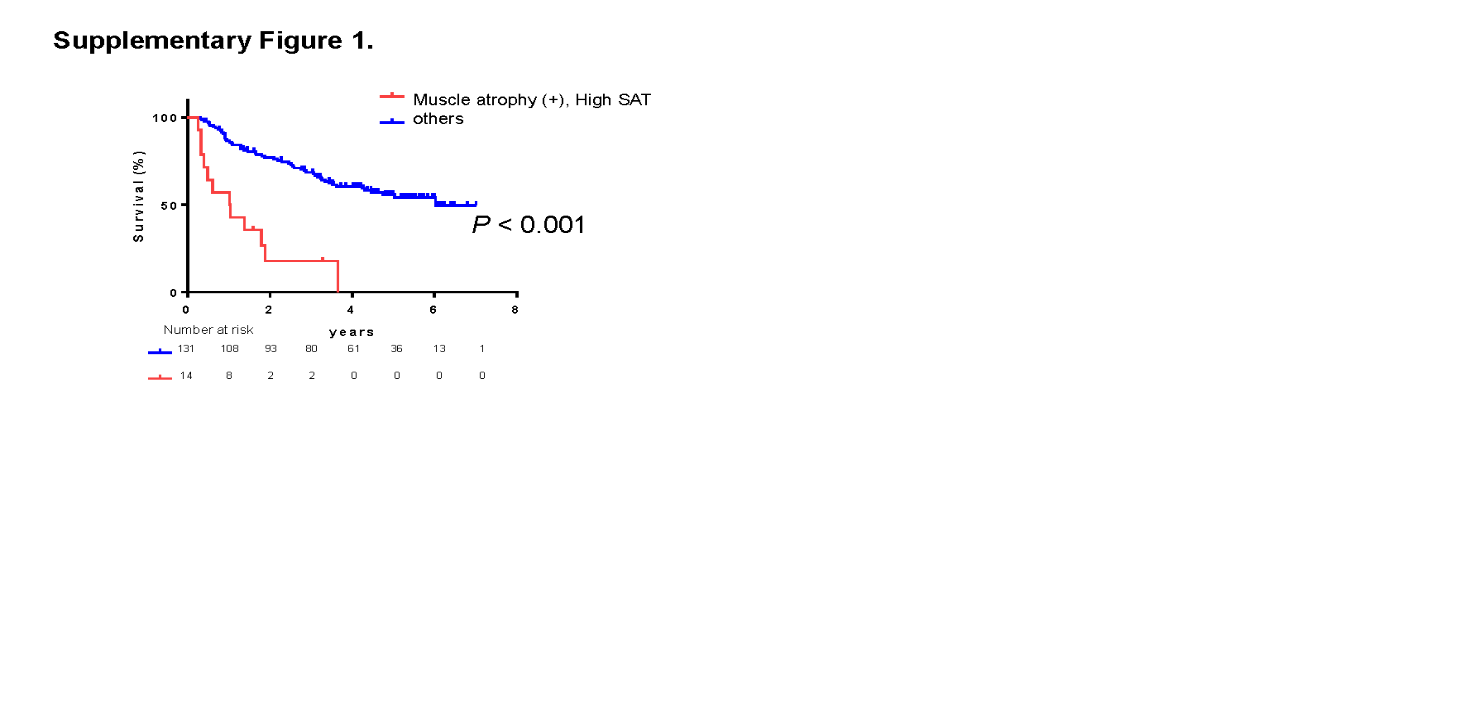


## Supplementary Figure 1. Comparison of overall survival between patients with both muscle atrophy and high SAT radiodensity and other patients. (A) Kaplan–Meier analysis was performed to stratify patients with and without muscle atrophy and high SAT radiodensity. The muscle mass was assessed by the simple method. The median overall survival is 1.03 years in patients with both muscle atrophy and high SAT radiodensity and 6.03 years in the other patients. Patients with both muscle atrophy and high SAT radiodensity have a poorer prognosis than the other patients (log-rank *P* < 0.001; crude Cox regression HR: 4.79, 95% CI: 1.48–15.5).

CI, confidence interval; HCC, hepatocellular carcinoma; HR, hazard ratio; SAT, subcutaneous adipose tissue.

## Supplementary Figure 2

**
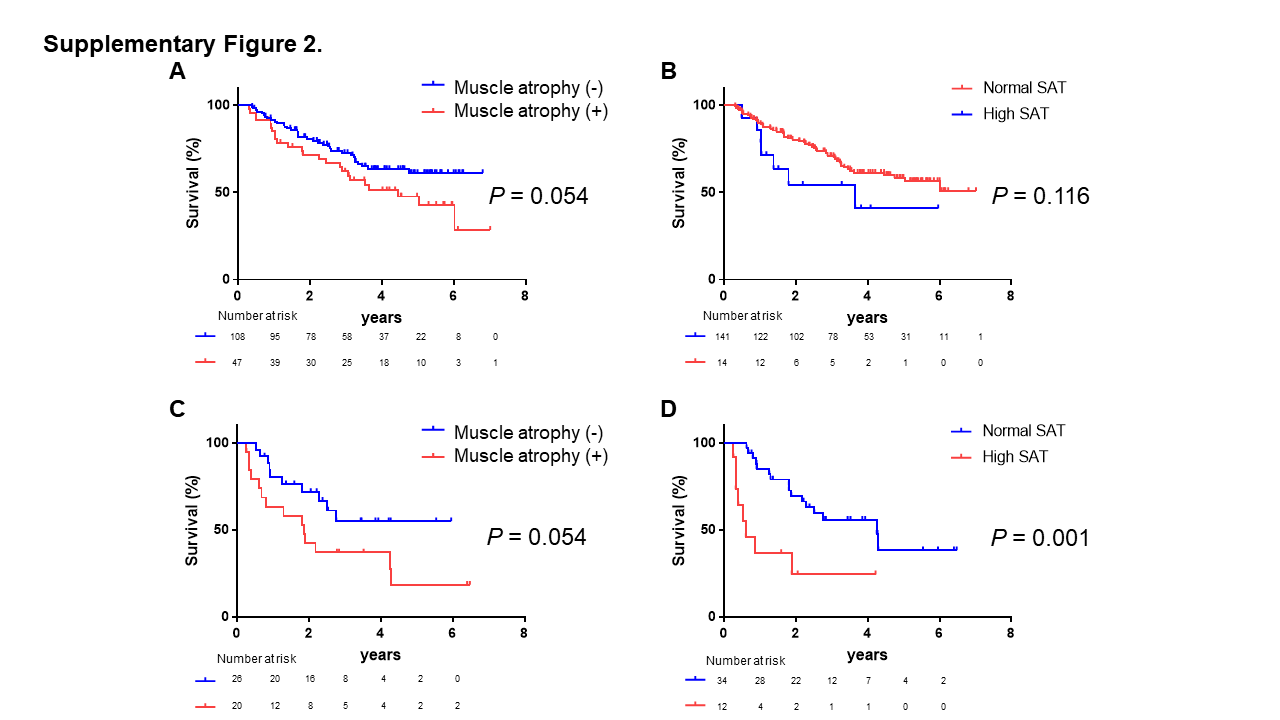
**

## Supplementary Figure 2. Survival rates in patients stratified according to Child–Pugh grade.

(A) Kaplan–Meier estimates of overall survival stratified according to muscle atrophy in HCC patients with Child–Pugh grade A. The median overall survival is not reached in patients without muscle atrophy and 4.46 years in patients with muscle atrophy. Patients with muscle atrophy tend to have a poorer prognosis compared to those without muscle atrophy (log-rank *P*-value = 0.054; crude Cox regression HR: 1.66, 95% CI: 0.95–2.90).

(B) Kaplan–Meier estimates of overall survival stratified according to SAT radiodensity in HCC patients with Child–Pugh grade A. The median overall survival is not reached in patients with normal SAT radiodensity and 3.65 years in patients with high SAT radiodensity. Patients with high SAT radiodensity tend to have a poorer prognosis compared to those with normal SAT radiodensity (log-rank *P*-value = 0.116; crude Cox regression HR: 1.86, 95% CI: 0.67–5.14).

(C) Kaplan–Meier estimates of overall survival stratified according to muscle atrophy in HCC patients with Child–Pugh grade B or C. The median overall survival is not reached in patients without muscle atrophy and 1.87 years in patients with muscle atrophy. Patients with muscle atrophy tend to have a poorer prognosis compared to those without muscle atrophy (log-rank *P*-value = 0.054; crude Cox regression HR: 2.17, 95% CI: 0.95–4.91).

(D) Kaplan–Meier estimates of overall survival stratified according to SAT radiodensity in HCC patients with Child–Pugh grade B or C. The median overall survival is 4.25 years in patients with normal SAT radiodensity and 0.61 years in patients with high SAT radiodensity. Patients with high SAT radiodensity have a significantly poorer prognosis than those with normal SAT radiodensity (log-rank *P*-value = 0.001; crude Cox regression HR: 3.61, 95% CI: 1.06–12.29).

CI, confidence interval; HCC, hepatocellular carcinoma; HR, hazard ratio; SAT, subcutaneous adipose tissue.

## Supplementary Figure 3


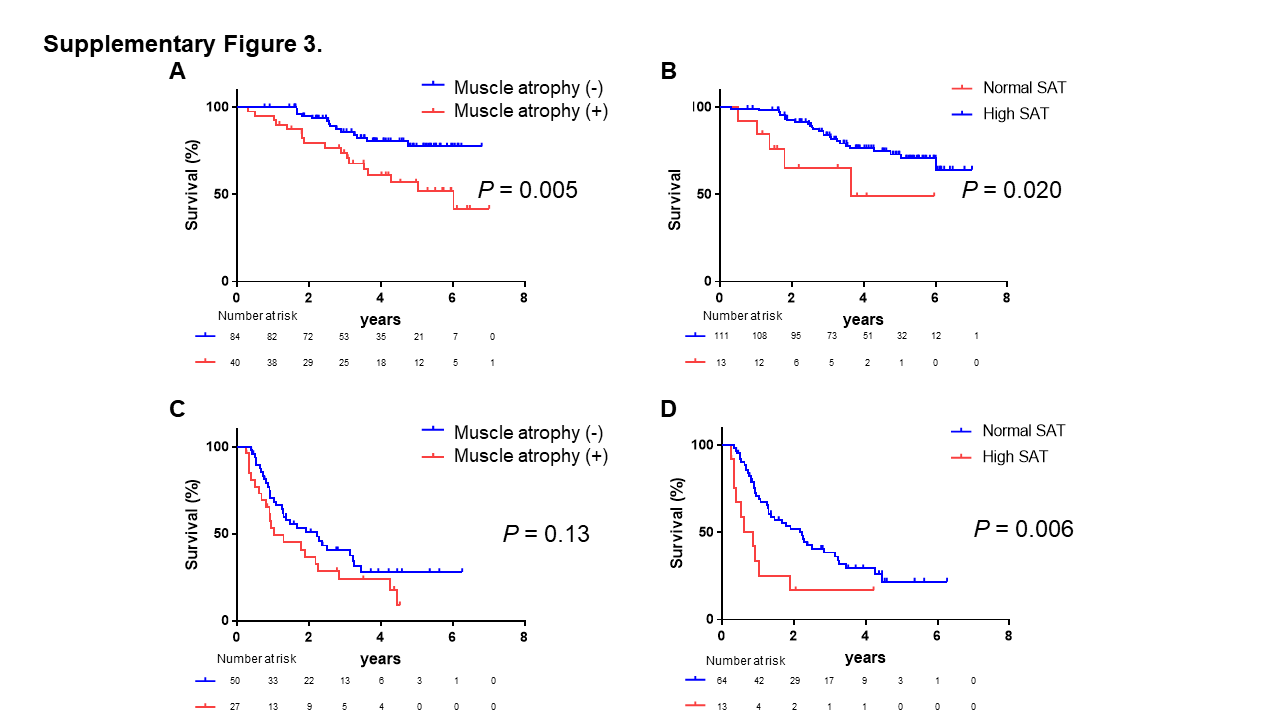


## Supplementary Figure 3. Survival rates stratified according to BCLC class.

(A) Kaplan–Meier estimates of overall survival stratified according to muscle atrophy in HCC patients with BCLC class 0 or A. The median overall survival is not reached in patients without muscle atrophy and 6.02 years in patients with muscle atrophy. Patients with muscle atrophy have a poorer prognosis than those without muscle atrophy (log-rank *P*-value = 0.005; Crude Cox Regression HR: 2.95, 95% CI: 1.38–6.30).

(B) Kaplan–Meier estimates of overall survival stratified according to SAT radiodensity in HCC patients with BCLC class 0 or A. The median overall survival is not reached in patients with normal SAT radiodensity and 3.65 years in patients with high SAT radiodensity. Patients with high SAT radiodensity have a poorer prognosis than those with normal SAT radiodensity (log-rank *P*-value = 0.020; crude Cox regression HR: 2.98, 95% CI: 1.13–7.84).

(C) Kaplan–Meier estimates of overall survival stratified according to muscle atrophy in HCC patients with BCLC class B or more. The median overall survival is 2.22 years in patients without muscle atrophy and 1.04 years in patients with muscle atrophy. Patients with muscle atrophy do not have a poorer prognosis than those without muscle atrophy (log-rank *P*-value = 0.13; crude Cox regression HR: 1.53, 95% CI: 0.84–2.75).

(D) Kaplan–Meier estimates of overall survival stratified according to SAT radiodensity in HCC patients with BCLC class B or more. The median overall survival is 2.18 years in patients with normal SAT radiodensity and 0.87 years in patients with high SAT radiodensity. Patients with high SAT radiodensity have a poorer prognosis than those with normal SAT radiodensity (log-rank *P*-value = 0.006; crude Cox regression HR: 2.59, 95% CI: 1.28–5.21).

BCLC, the Barcelona Clinic Liver Cancer; CI, confidence interval; HCC, hepatocellular carcinoma; HR, hazard ratio; SAT, subcutaneous adipose tissue.

## Supplementary Figure 4

**
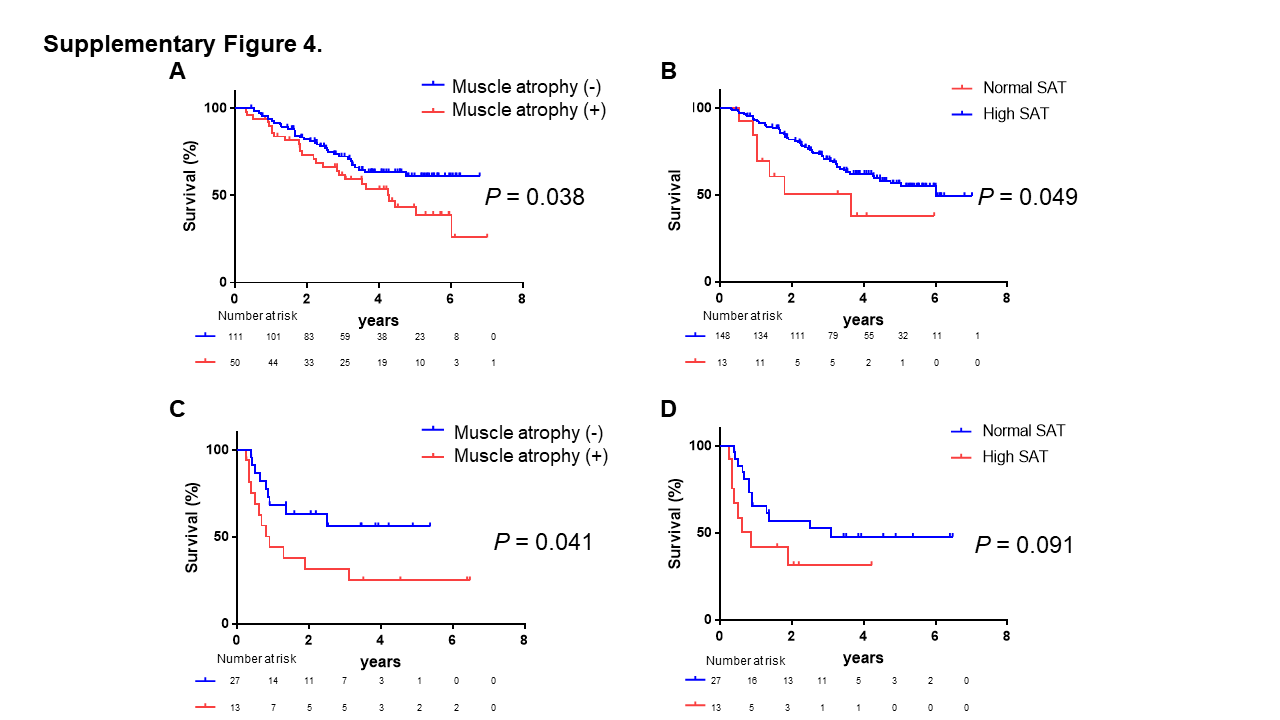
**

## Supplementary Figure 4. Survival rates stratified according to fluid retention.

(A) Kaplan–Meier estimates of overall survival stratified according to muscle atrophy in HCC patients without fluid retention. The median overall survival is not reached in patients without muscle atrophy and is 4.30 years in patients with muscle atrophy. Patients with muscle atrophy have a poorer prognosis than those without muscle atrophy (log-rank *P*-value = 0.038; crude Cox regression HR: 1.70, 95% CI: 1.02–2.81).

(B) Kaplan–Meier estimates of overall survival stratified according to SAT radiodensity in HCC patients without fluid retention. The median overall survival is 6.02 years in patients with normal SAT radiodensity and 3.65 years in patients with high SAT radiodensity. Patients with high SAT radiodensity have a poorer prognosis than those with normal SAT radiodensity (log-rank *P*-value = 0.049; crude Cox regression HR: 2.99, 95% CI: 1.01–8.88).

(C) Kaplan–Meier estimates of overall survival stratified according to muscle atrophy in HCC patients with fluid retention. The median overall survival is not reached in patients without muscle atrophy and 0.90 years in patients with muscle atrophy. Patients with muscle atrophy have a poorer prognosis than those without muscle atrophy (log-rank *P*-value = 0.041; crude Cox regression HR: 2.40, 95% CI: 1.01–5.70).

(D) Kaplan–Meier estimates of overall survival stratified according to SAT radiodensity in HCC patients without fluid retention. The median overall survival is 3.10 years in patients with normal SAT radiodensity and 0.87 years in patients with high SAT radiodensity. Patients with high SAT radiodensity have a poorer prognosis than those with normal SAT radiodensity (log-rank *P*-value = 0.091; crude Cox regression HR: 2.08, 95% CI: 0.75–5.77).

CI, confidence interval; HCC, hepatocellular carcinoma; HR, hazard ratio; SAT, subcutaneous adipose tissue.

# Supplementary Method

## Skeletal muscle mass calculation by computed tomography imaging methods

For the simpler skeletal muscle mass calculation in computed tomography (CT) imaging, the following simple CT method was employed: the left–right sum of the long axis was multiplied by the short axis of the iliopsoas muscles at the level of L3 and the product was divided by the height squared.
